# Effective and ecological half-lives of ^90^Sr and ^137^Cs observed in wheat and rice in Japan

**DOI:** 10.1007/s10967-015-4352-6

**Published:** 2015-08-09

**Authors:** Stefan Merz, Katsumi Shozugawa, Georg Steinhauser

**Affiliations:** Vienna University of Technology, Atominstitut, Stadionallee 2, 1020 Vienna, Austria; Graduate School of Arts and Sciences, The University of Tokyo, Meguro-ku, Tokyo, Japan; Environmental and Radiological Health Sciences, Colorado State University, Fort Collins, CO 80523 USA; Institute of Environmental Radioactivity, Fukushima University, Fukushima, 960-1296 Japan; Leibniz Universität Hannover, Institut für Radioökologie und Strahlenschutz, 30419 Hannover, Germany

**Keywords:** Fukushima, Foodstuff, Food safety, Effective half-life, Ecological half-life, ^90^Sr, ^137^Cs

## Abstract

Published pre-Fukushima food monitoring data from 1963 to 1995 were used to study the long-term presence of ^137^Cs and ^90^Sr in rice and wheat. Effective half-lives (*T*_eff_) were calculated for rice (^137^Cs: 5.6 years; ^90^Sr: 6.7 years) and wheat (^137^Cs: 3.5 years; ^90^Sr: 6.2 years), respectively. In rice, ^137^Cs exhibits a longer *T*_eff_ because putrefaction processes will lead to the formation of NH_4_^+^ ions that are efficient ion exchangers for mineral-adsorbed cesium ions, hence making it more readily available to the plant. Knowledge on the long-term behavior of radiocesium and radiostrontium will be important for Japanese food-safety campaigns after the Fukushima nuclear accident.

## Introduction

Nuclear accidents cause an array of health problems, both physical and mental. Fear-related problems are among the most underestimated consequences after a severe nuclear accident such as Chernobyl or Fukushima [1–3]. The lack of a sensory organ is one major reason for doubts about the own safety [4], especially when consuming potentially contaminated food. Indeed, the intake of contaminated food is the most severe pathway for exposure after nuclear accidents [5–8], which also potentially affects remote communities that have not been affected by direct fallout from the accident, but may consume contaminated food that is transported from contaminated regions.

In the aftermath of the Fukushima nuclear accident, the Japanese authorities have put much effort into a food monitoring program that stands unprecedented in human history [9]. More than 900,000 samples have been measured to date. This campaign especially also focused on rice that is traditionally a very important food item in Japanese cuisine [10]. These efforts have been supported by whole body counting campaigns of children and adult residents [11] as well as food duplicate studies [12, 13].

From a radioecological point of view, the behavior of radionuclides in the environment and the interaction (uptake, excretion) with food organisms (plants, fungi, animals) is essential for the prediction of future internal exposure due to intake of contaminated foods. Fungi, for example, have long been known to hyper-accumulate radiocesium from soil, leading to remarkable activities in certain mushroom species (and animals that feed on them). The presence and availability to the plant of a radionuclide depends on its physical half-life and its ecological half-life that is determined by wash-out and immobilization of the nuclide. The objective of this study was to determine the effective and ecological half-lives of ^137^Cs and ^90^Sr in both rice and wheat in Japan, as taken up from soil.

The effective half-life (*T*_eff_) combines both the physical decay as well as ecological “losses” such as migration or immobilization by adsorption on soil minerals. The ecological losses are expressed by the ecological half-life (*T*_eco_). The correlation between physical half-life (*T*_1/2_), *T*_eff_, and *T*_eco_ is shown in Eq. 1 [14].

1 $$\frac{1}{{T_{\text{eff}} }} = \frac{1}{{T_{\text{eco}} }} + \frac{1}{{T_{1/2} }}.$$

## Materials and methods

This study is based on pre-Fukushima (1959–1995) ^90^Sr and ^137^Cs data for polished rice [15] and polished wheat [16] that were published by Komamura et al. who studied the effect of the 20th century nuclear weapons fallout. This unique data set allows for the study of the long-term behavior of both radionuclides. What makes both papers especially valuable is that they report on the activity concentrations in rice and wheat in *Japan* (thus ideally representing the environmental setting that will be relevant for post-Fukushima challenges) and in *multiple samples* per year (thus representing a credible average). Due to its volcanic history, Japanese soil (acidic soil) is much distinct from non-volcanic soil and exhibits a unique soil chemistry. Like clay minerals, high-silica volcanic ash exhibits cation exchange properties [17].

The focus of the present study was on both radionuclides ^137^Cs (*T*_1/2_ = 30.2 years) as well as the understudied radionuclide ^90^Sr (*T*_1/2_ = 28.6 years) [18, 19]. We analyzed the data of nationwide averages per year published by Komamura et al. [15, 16].

## Results and discussion

The source of ^137^Cs and ^90^Sr in food (after uptake by plants from soil) has been dominated for a long time by the fallout of atmospheric nuclear explosions. Nuclear accidents, however, in particular Chernobyl and Fukushima have the potential to massively increase the fallout-background locally and regionally. In Austria, for example, the ^137^Cs inventory in soil is approximately 10 % due to weapons fallout and 90 % due to the Chernobyl accident [20, 21]. Continuous input from ongoing fallout caused by the cold war’s weapons race in the late 1950s and early 1960s led to a constantly increasing environmental inventory of ^90^Sr and ^137^Cs. A constantly increasing contamination of soil does not allow for the establishment of a certain, stable level which makes environmental processes establishing the ecological half-life observable. Therefore, we decided to neglect data prior to 1963. In 1963, the Partial Nuclear Test-Ban Treaty was signed that prohibited atmospheric nuclear tests. In this year, the activity levels in the data base also reached a maximum for both ^90^Sr and ^137^Cs. It appeared reasonable to calculate the *T*_eff_ and *T*_eco_ from this maximum value, similar to our previous investigation of the apparent half-life of ^131^I in animal thyroids [22].

**Fig. 1:**
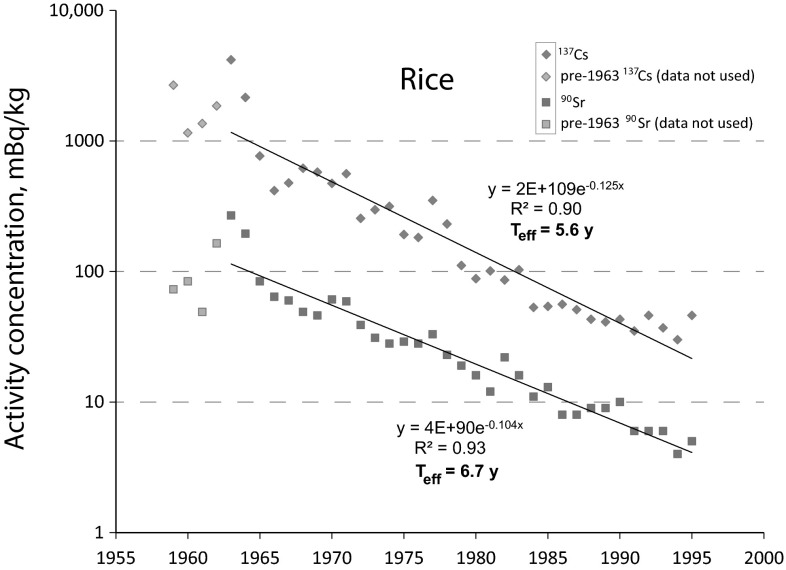
Activity concentrations of ^90^Sr and ^137^Cs in rice and their development over time (1959–1995) [15]. Only data from 1963 onwards were used for calculation of the exponential trend line

**Fig. 2:**
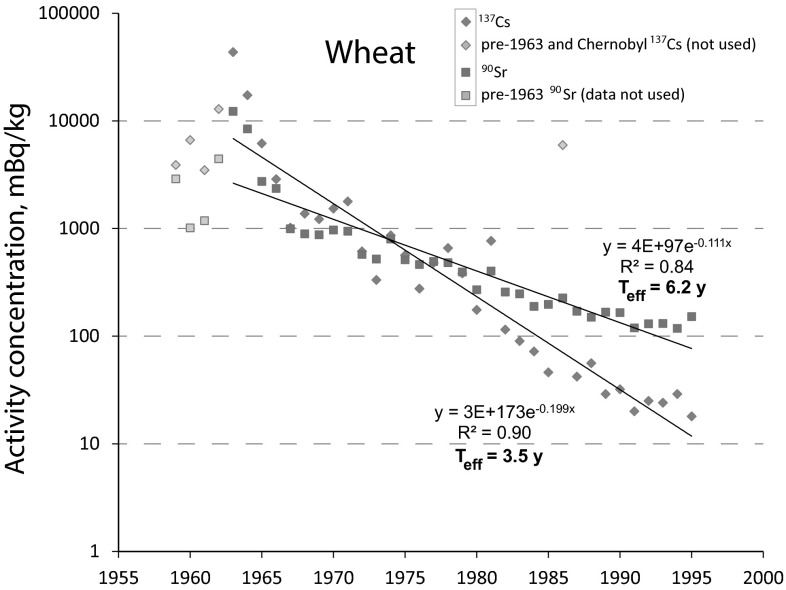
Activity concentrations of ^90^Sr and ^137^Cs in wheat and their development over time (1959–1995) [16]. Only data from 1963 onwards were used for calculation of the exponential trend line. For ^137^Cs, the data point from 1986 (Chernobyl) was also rejected as it represents an obvious outlier in the data set

The results of the calculations are illustrated in Fig. 1 (rice) and Fig. 2 (wheat). For the exponential trend line, only data from 1963 were used for the above reason. In the data set for ^137^Cs in wheat (interestingly not in rice), an obvious outlier was observable in 1986—the year of the Chernobyl accident. Rejecting this data point greatly improved the coefficient of determination (*R*^2^) from 0.77 to 0.90. Rejecting the 1986 ^90^Sr data point in rice did not have any effect on the *R*^2^. This is in line with many previous observations showing that the Chernobyl nuclear accident has been a much more powerful source of ^137^Cs than of ^90^Sr [1]. Even for ^137^Cs, the following years after Chernobyl proved to follow the pre-Chernobyl pattern and hence were included in the calculation. It appears likely that, due to the long distance and maximum ^137^Cs activity concentrations in air of “only” 0.074 Bq/m^3^ in Japan [23], the Chernobyl nuclear accident did not significantly increase the radiocesium inventory in soil in Japan. Instead, it can be speculated that foliar uptake by the wheat plant and direct deposition of ^137^Cs particulates on the wheat grains has increased the ^137^Cs activity concentration in wheat in 1986 only.

The effective half-lives were derived from the decay constants in Figs. 1 and 2, respectively. For rice, the effective half-lives were *T*_eff_(^137^Cs) = 5.6 years and *T*_eff_(^90^Sr) = 6.7 years, respectively. In wheat, the effective half-lives were *T*_eff_(^137^Cs) = 3.5 years and *T*_eff_(^90^Sr) = 6.2 years, respectively.

From Eq. 1, the ecological half-lives were derived. In rice, they were *T*_eco_(^137^Cs) = 6.8 years and *T*_eco_(^90^Sr) = 8.7 years, respectively. In wheat, the ecological half-lives were *T*_eco_(^137^Cs) = 3.9 years and *T*_eco_(^90^Sr) = 8.0 years, respectively.

While the *T*_eff_ of ^90^Sr are comparable for both wheat and rice, it is interesting to note that the *T*_eff_ of ^137^Cs is significantly shorter for wheat than for rice. The main reason for this discrepancy is most probably the agricultural production technique in rice paddies. Putrefaction processes under water cause the formation of ammonia (NH_3_) that readily dissolves in water, thus forming ammonium (NH_4_^+^) ions. These ammonium ions have been identified previously [24] as powerful ion exchangers for Cs^+^ ions adsorbed on (clay) minerals. Other reasons for explaining this discrepancy may be different plant-related uptake mechanisms and kinetics for Sr^2+^ and Cs^+^, respectively and different translocation inside the plant.

After the Fukushima nuclear accident, the authorities assumed an intrinsic coexistence of ^137^Cs and ^90^Sr and thus a constant ^90^Sr/^137^Cs ratio in foods. The maximum contribution of ^90^Sr was assumed to be ≤10 % (after April 2012 ≤0.3 %) of the respective ^137^Cs activity concentration in food. This allowed for the rapid determination of ^137^Cs using gamma-spectrometry and the subsequent estimation of the dose contribution caused by ^90^Sr (a radionuclide that requires a very laborious and time-consuming separation prior to radiometric analysis). In our previous study [9] we discovered that the assumption of a constant ^90^Sr/^137^Cs ratio is not justified over longer periods of time. In agreement with our previous conclusions we can now specify that the different effective ecological half-lives of both radionuclides thwart the assumption of a constant ratio over time, thus potentially putting the reliability and public credibility of the food monitoring program at stake.

## Conclusions

We presented evidence for significantly different effective half-lives of ^137^Cs (3.5 years) and ^90^Sr (6.2 years) in wheat, which will be of great importance for food safety considerations. In rice, in contrast, the effective half-lives of ^137^Cs (5.6 years) and ^90^Sr (6.7 years) proved to be rather comparable, which can probably be explained by exceptional properties of the agricultural production technique in a paddy. This technique, in particular, the formation of NH_4_^+^ ions (superior ion exchangers), makes radiocesium more bioavailable to the rice plant compared to the wheat plant. The longer *T*_eff_ of ^137^Cs observed in rice hence should be considered as an ecological exception and should not be applied on other food items without experimental evidence. The ecological half-lives in rice were *T*_eco_(^137^Cs) = 6.8 years and *T*_eco_(^90^Sr) = 8.7 years, respectively. For wheat, *T*_eco_(^137^Cs) = 3.9 years and *T*_eco_(^90^Sr) = 8.0 years were calculated. In both rice and wheat, the ecological half-lives of ^90^Sr were longer compared to ^137^Cs, which is most likely due to the higher bioavailability of strontium in soil, because cesium ions have a higher affinity to clay minerals and become immobilized more readily.
